# Latex balloons: an alternative, low-cost model for vascular anastomosis training in medical education

**DOI:** 10.1590/1677-5449.170111

**Published:** 2018

**Authors:** Priscilla Lopes Fonseca Abrantes Sarmento, André Loureiro Fernandes, Bruna Lisboa do Vale, Bruno D’Paula Andrade, Jennyfer Kellen Lázaro da Rocha, Jéssika da Silva Antas, Waleria Cristina de Abreu, Petrúcio Abrantes Sarmento

**Affiliations:** 1 Universidade Federal da Paraíba – UFPB, Departamento de Cirurgia, João Pessoa, PB, Brasil.

**Keywords:** education, medical, undergraduate, vascular surgical procedures, anastomosis, surgical, models, anatomic

## Abstract

Simulators are increasingly being used in medical education, but accessibility is restricted by their elevated cost. A accessible and low-cost model was developed for teaching and learning vascular sutures and anastomoses at a Basic Surgical Techniques Laboratory. Latex balloons of varying colors, polypropylene 6.0 sutures, and other materials specifically for suturing (needle holder and forceps) were used. The balloons were fixed to screws inserted into wooden boards in order to facilitate repairs. E end-to-end, end-to-side, and side-to-side anastomoses and patching were performed. Anastomosis patency was tested by injecting water into one extremity of the balloon and observing the liquid exit via the opposite extremity. The advantages observed with this training model for anastomoses were malleability, resistance to passage of the suture, and the fact that it is inorganic. Latex balloons are an inexpensive option that are non‑perishable and offer prolonged use for teaching and practice of arterial sutures and anastomoses.

## INTRODUCTION

 At a Brazilian university, fourth-semester medical students on the Basic Surgical and Anesthetic Techniques course start to learn about surgical materials, such as instruments and threads, and about surgical techniques, such as knots, sutures, and anastomoses. A self-assessment in which students, monitors and teachers evaluated their learning and teaching in the classes and a course assessment questionnaire completed by the medical students revealed deficiencies in theoretical and practical teaching on the subject of anastomoses. We therefore proposed that this subject should be dealt with in greater depth, starting with vascular anastomoses, because of the importance of this type of anastomosis to all surgical specialties and not just to vascular or cardiovascular surgery. 

 Simulators are being used with increasing frequency in medical training, but they are not accessible to the majority of medical schools and hospitals in Brazil because of the elevated cost. There are descriptions in the literature of using synthetic materials, such as silicone and rubber gloves, animal tissues, and vegetable tissues, and of gaining practical experience using small animals (pigs, rats, and rabbits), as part of teaching of vascular anastomosis and development of the skills needed. 1 ^-^ 5 However, use of animal models is the most common objection raised by the public and by the students themselves, who question the practice of sacrificing animals for educational purposes. 6 The strict rules mandated by the Institutional Committees on Care and Utilization of Animals also make using such animal models in medical training difficult, contributing to the need to develop alternatives. 

 The objective of this article was to describe the process of creation of a model using latex balloons for teaching and practicing vascular anastomoses and sutures in the laboratory of the Basic Surgical Techniques course at a Brazilian University. 

## MATERIALS AND METHODS

**Figure 1:**
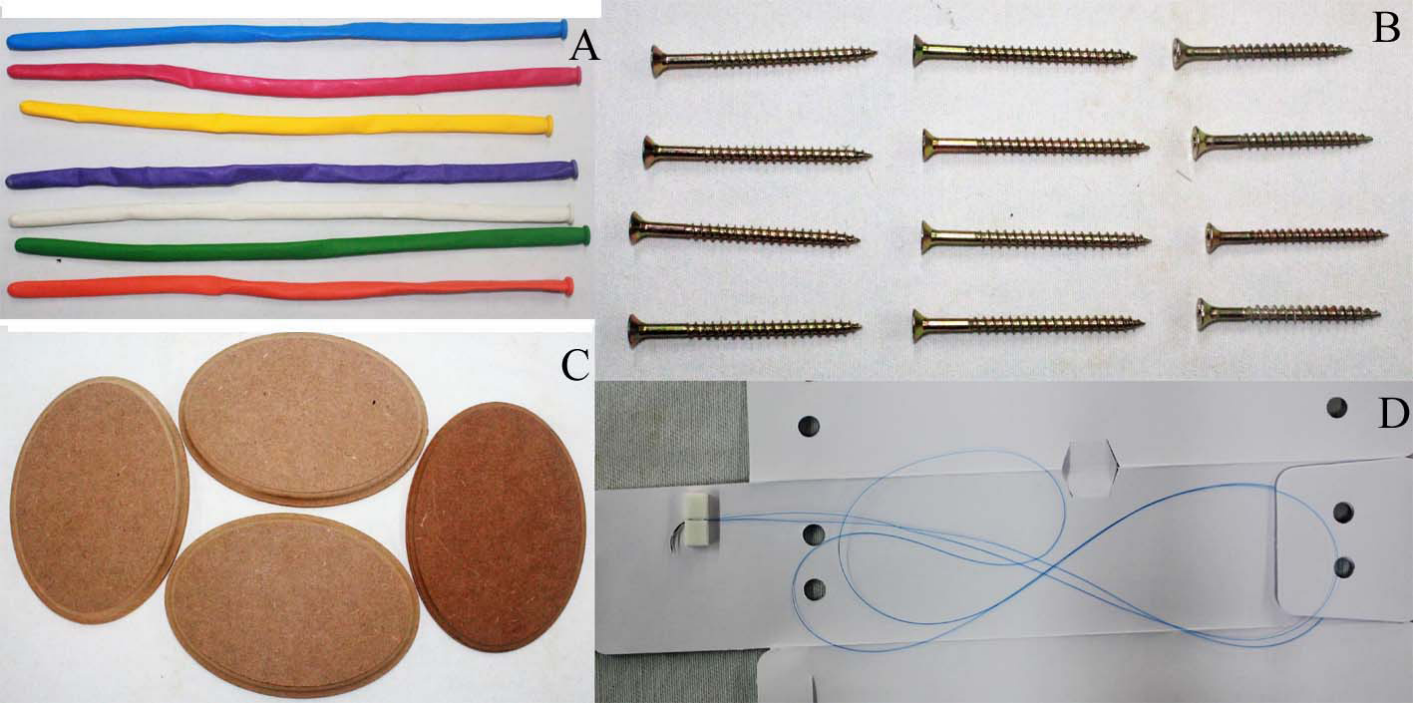
Materials used to construct the model: latex balloons in several different colors (A); woodscrews (B); wooden boards (C); 6.0 polypropylene sutures and two needles (D).

 The following materials were used to construct the model: seven 27 x 0.9 cm latex balloons of varying colors, four wooden boards, nine woodscrews, polypropylene cardiovascular 6.0 surgical sutures, two 1.3 cm needles ( Figure 1 ) and other materials specifically for vascular sutures (needle holder, Kelly forceps with latex protection, and non-traumatic clamp forceps). 

**Figure 2:**
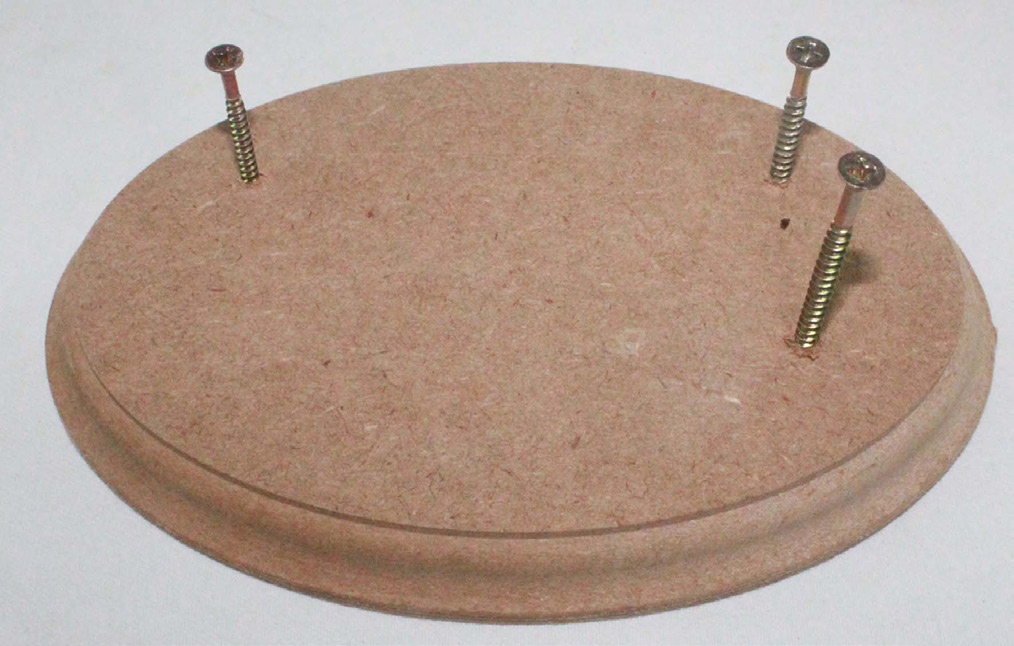
Preparation of a wooden board with woodscrews.

 The extremities and excess length of the balloons were pulled down over the screws ( Figure 2 ). This enabled the model top be fixed to the wooden board and saved material, since the excess length that had been furled over the screw rather than discarded could be used later. 

 The sutured patching and anastomoses were performed on balloons in accordance with the basic principles of vascular anastomoses described by Carrel, 7 ^-^ 9 Guthrie, 10 and Rutherford, 11 using two polypropylene sutures and two needles in each case. 

**Figure 3:**
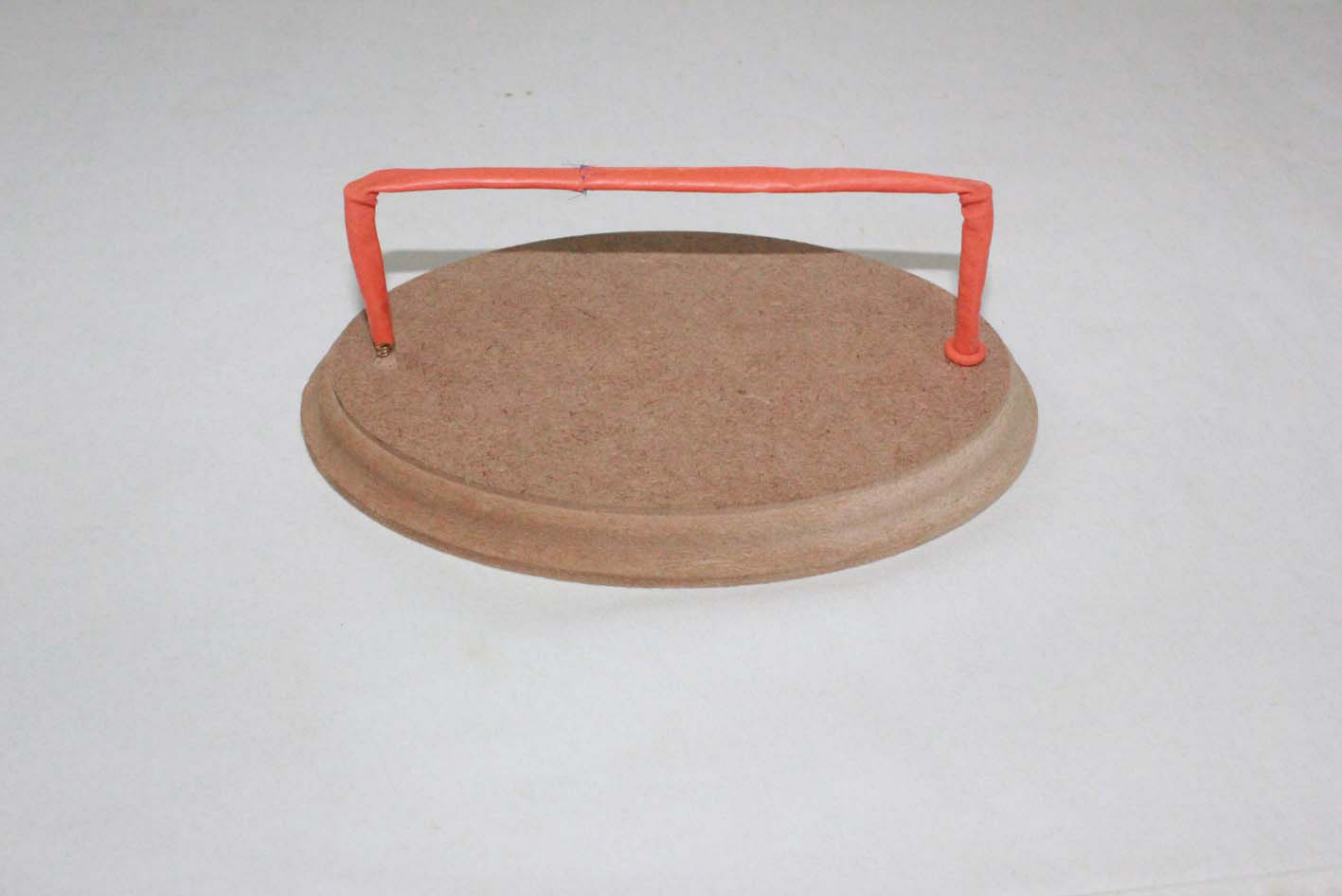
End-to-end anastomosis with interrupted stitches.

 End-to-end anastomosis was performed with interrupted stitches ( Figure 3 ) using the triangulation technique, 12 ^,^ 13 with three equidistant stitches, transforming the circumference into a triangle. This type of anastomosis is recommended for children and smaller caliber vessels. Since both extremities were of the same caliber, they were not beveled. Using this technique, the edges can also be drawn together using simple running sutures. 

**Figure 4:**
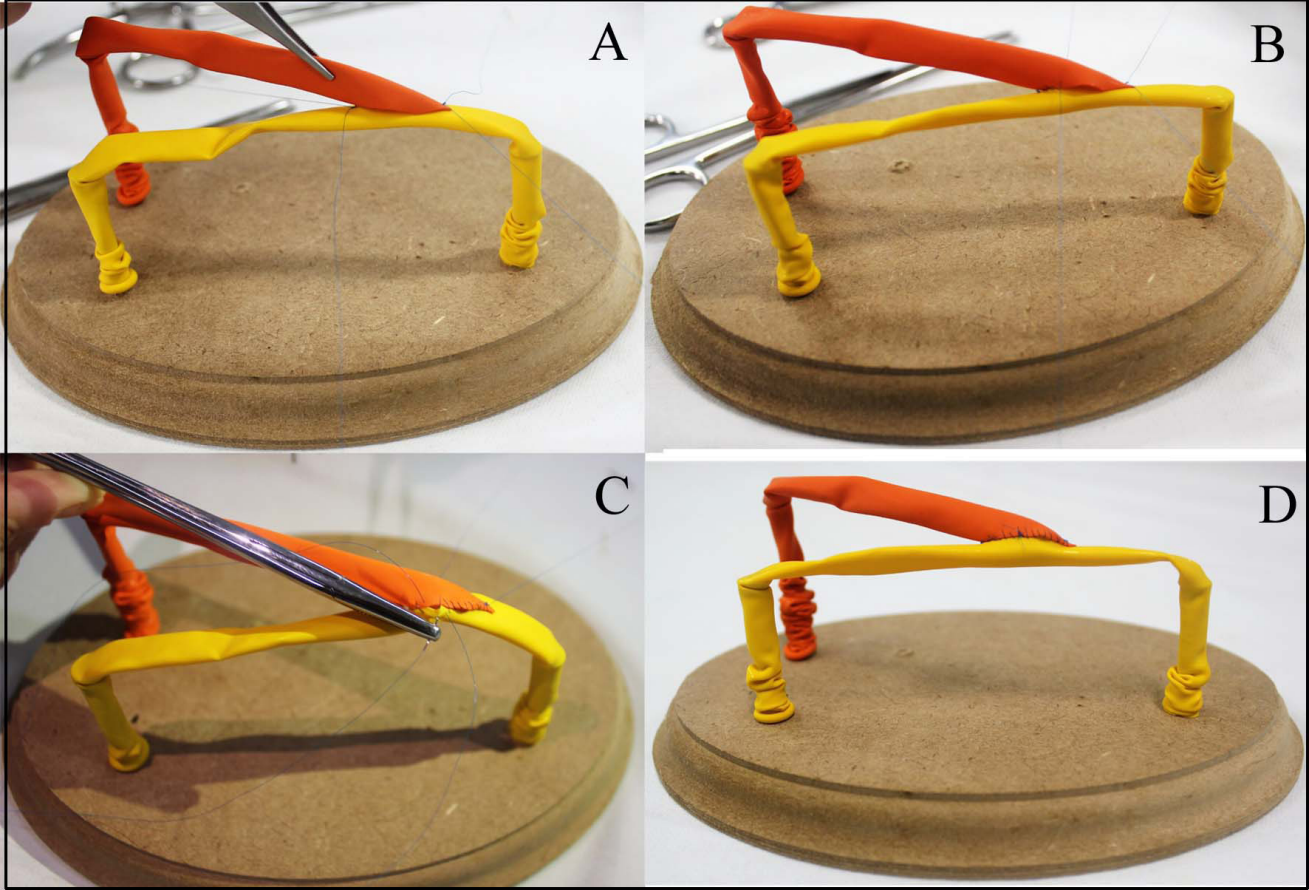
Performing an end-to-side anastomosis: initial suture drawing the balloons together with stitches at the angles (proximal and distal) (A); continuous sutures in four quadrants (B); detail of needle pulled entirely through the wall of the balloon (C); final appearance of the anastomosis (D).

 The patch suture and the end-to-side and side-to-side anastomoses were conducted by placing initial stitches at the angles and then closing the anterior and posterior walls with continuous sutures. 12 ^,^ 13 Additionally, for the end-to-side anastomosis, using the technique recommended by Rutherford, the side opening in the balloon was made one and a half times the caliber of the balloon to be implanted and beveled at a 30 to 45 degree angle in relation to the same balloon. Some of the stages involved in the end-to-side anastomosis are illustrated in Figure 4 . 

**Figure 5:**
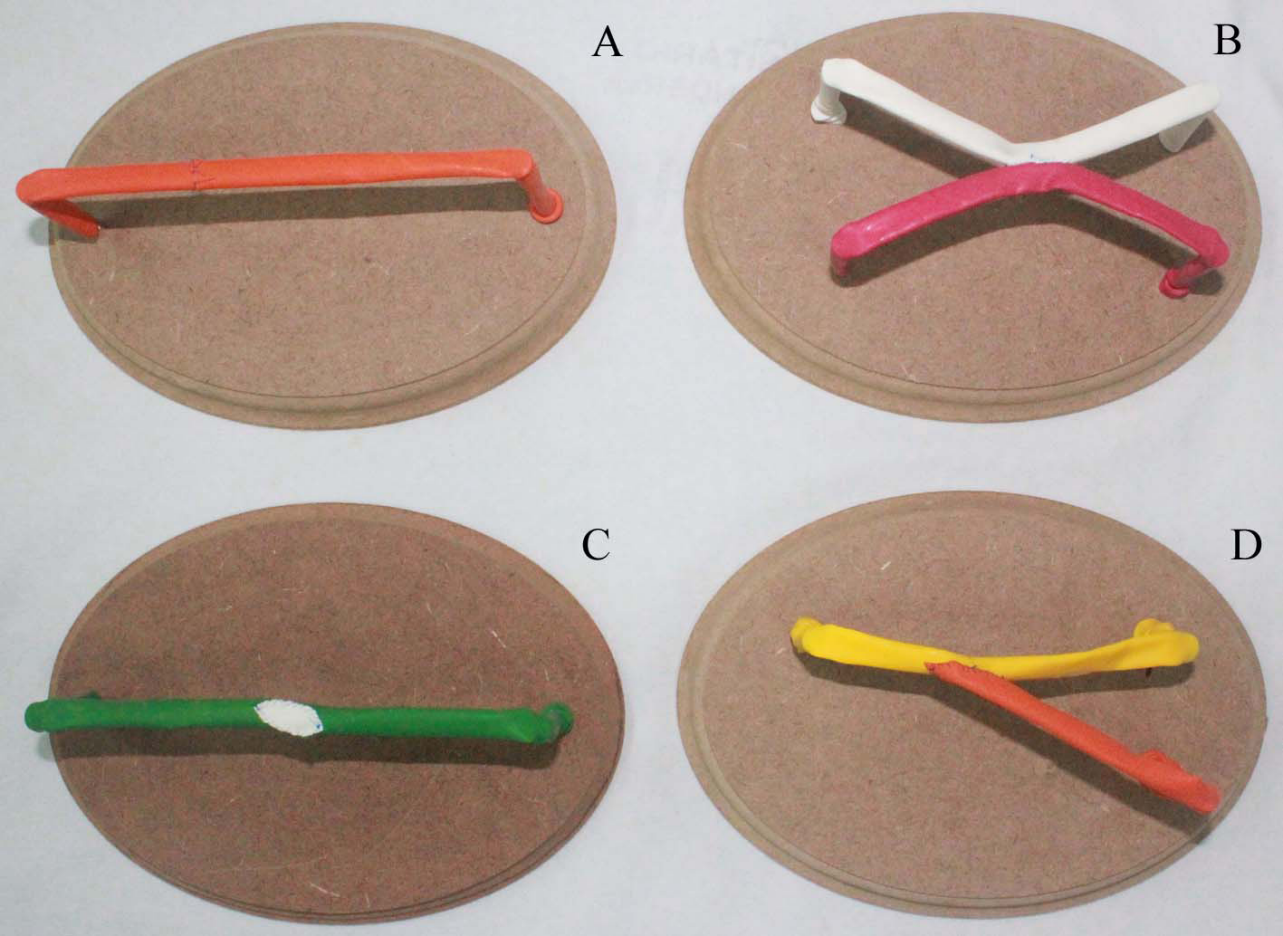
Final appearance of the anastomosis models on the four wooden boards: end-to-end (A); side-to-side (B); patch (C) and end-to-side (D).

 Thus, having constructed the model, several types of anastomoses were tested: end-to-end, side-to-side, and end-to-side anastomoses, and patch construction, using four different wooden boards, with final appearance as shown in Figure 5 . 

 Patency of the patched balloon and the anastomoses was tested by injecting water via one extremity of each balloon and observing it exiting via the opposite extremity (end-to-end and side-to-side anastomoses and patch) or the extremity of interest (end-to-side anastomosis). 

## RESULTS

 Vascular anastomoses (end-to-end, side-to-side, end-to-side) were conducted on three different models, and the patch was constructed on a fourth. Different colored latex balloons were used for each “vessel”. When suturing, resistance to passage of the needle through the latex balloon was observed, similar to that observed in normal, non-atherosclerotic arteries and veins. The material is firm, and no lacerations occurred in any of the models. Additionally, the edges remained well attached by the threads and knots. The different colors of each balloon used for the anastomoses is an interesting feature, since it facilitates visualization of the sutures passing through the material, the edges being drawn together, and the end result. 

 Patency was 100%, as tested by injection of liquid. In the end-to-end anastomosis and patch models, liquid was also observed leaking between stitches. 

 The total cost of construction of the model, including the items used for the four anastomoses, was R$ 152.00 (eight polypropylene 6.0 surgical sutures, seven latex balloons, woodscrews, and four wooden boards), the majority of which was spent on purchasing the surgical sutures. 

## DISCUSSION

 Vascular anastomosis construction is a procedure that is conducted in many surgical specialties and is not restricted to vascular and cardiovascular surgery. 14 ^,^ 15 In the majority of cases, students and trainee surgeons learn and practice anastomoses directly on human beings, which can cause harm to patients because of increased duration of surgery or postoperative complications, and consequently increase hospital costs. 14 ^,^ 16 In many cases, this practice is the result of routines that have become established in services over many years, or because of a lack of laboratories equipped with accessible, synthetic models at medical schools or in non-hospital settings. 

 Several different studies have already demonstrated that both teaching and practice should be conducted in laboratories before they are attempted in a hospital environment or in clinical practice. 14 ^,^ 15 ^,^ 17 Some authors recommend that preliminary training on simulators should be a basic stage in the training of future surgeons. 2 ^,^ 18 In response to the elevated cost of commercially-available simulators, a number of low-cost alternatives have been developed. 

 Excluding use of live animals, the literature contains descriptions of several types of models using a variety of materials. 

 Webster & Ely 18 and Lima et al. 19 tested end-to-end anastomosis constructed using mononylon suture thread and silicone tubes, showing that this is an effective technique for initial training. 

 Some authors mention using rubber gloves for vascular anastomosis practice, but they do not provide bibliographic references or descriptions of the methods and results. 

 Achar et al. 1 described an experimental model with the trachea and esophagus extracted from a chicken head and used to simulate arterial suturing to construct an end-to-end anastomosis. Colpan et al. 20 developed a realistic model for practicing vascular anastomoses using carotids from turkey necks, which were perfused during the procedure. Maluf et al. 5 used the vascular pedicle of spleens from post morten pigs that had undergone splenectomy as an alternative model for training vascular anastomosis with mononylon suture. 

 Grahem et al. 2 tested green beans and yardlong beans as low-cost models for end-to-end anastomosis with polypropylene suture and considered that the characteristics of green beans were more appropriate for initial training. 

 The material used in our study, latex balloons, has not previously been described in the literature for this purpose. The latex balloons are similar to vessels because they are cylindrical (27 cm long), have a caliber of 0.9 cm, a thin wall (less than 1 mm), and an internal lumen, and are flexible. Additionally, they also have similarities to the walls of normal arteries and veins since they provide little resistance to the passage of the needle with polypropylene suture attached. 

 The characteristics of latex, in terms of malleability and resistance, are favorable for teaching techniques for the various different types of anastomosis (end-to-end, side-to-side, end-to-side, and patching) that we tested in our study. We demonstrated that the principal advantage of the model using latex balloons is the possibility of using it to teach several different vascular suture techniques, in contrast to what is described in the majority of studies in the literature, which only tested end-to-end anastomosis. 2 ^,^ 3 ^,^ 6 ^,^ 19 ^,^ 20 Some studies used mononylon sutures to perform anastomoses. 6 ^,^ 19 ^,^ 20 In our model, anastomoses were constructed using fine polypropylene sutures and two needles, to make the model more realistic and help familiarize the students with the vascular technique. 

 Another notable characteristic is the different colors of the balloons employed, facilitating visualization of the parts to be anastomosed, which is relevant to students’ learning and understanding of anastomosis construction. Like silicone, latex is an inorganic material that is accessible and widely available, which are useful characteristics and become essential at centers that, like ours, do not have an animal house or an experimental surgery laboratory. 

 Two disadvantages with our model were observed. The first was the absence of an intimal layer in the balloon. When conducting vascular anastomoses in vivo, care is taken with attachment and with maintenance of the connection between the tunica intima and the wall of the vessel, in order to avoid detachment, or “flapping”. Problems involving this layer are often predisposing factors in postoperative complications and occlusions of vascular anastomoses. The second was leakage of the liquid injected to test the sutures, making the model unsuitable for assessing adequacy of the distance between stitches. It is believed that this was the result of the intrinsic characteristics of the balloon – being malleable, elastic, and inorganic – and the absence of blood coagulation factors that provoke cohesion of tissue cells. The same disadvantages were also observed by Grahem et al. 2 in models using vegetable tissues. 

 However, we believe that during the initial learning curve of students, and even young surgeons, the advantages outweigh the disadvantages. This is especially true with regard to teaching the technique, to training and familiarization with specific vascular instruments, to practicing anastomosis of structures using very fine sutures and two needles, and to progressive development of the technical dexterity and swiftness needed in combination with delicate movements. 

## CONCLUSIONS

 Latex balloons are an interesting option, that are feasible, and inexpensive in comparison to previous models described in the literature for teaching the technique and practicing sutures and the several different types of vascular anastomoses by medical students and novice surgeons. Their characteristics make them universally available and accessible, contributing to improving medical education. 
