# Supplementary material for: Latex balloons: an alternative, low-cost model for vascular anastomosis training in medical education
Source: J Vasc Bras. 2018 Jul-Sep;17(3):267–72. doi: 10.1590/1677-5449.170111 (PMC6326138; doi:10.1590/1677-5449.170111)
Supplement: Translation

# Translation: Balões de látex: um modelo alternativo e de baixo custo para treinamento de anastomoses vasculares no ensino médico

**Authors:** Priscilla Lopes Fonseca Abrantes Sarmento, André Loureiro Fernandes, Bruna Lisboa do Vale, Bruno D’Paula Andrade, Jennyfer Kellen Lázaro da Rocha, Jéssika da Silva Antas, Waleria Cristina de Abreu, Petrúcio Abrantes Sarmento

**Affiliations:** 1 Universidade Federal da Paraíba – UFPB, Departamento de Cirurgia, João Pessoa, PB, Brasil.

**Keywords:** educação de graduação em medicina, procedimentos cirúrgicos vasculares, anastomose cirúrgica, modelos anatômicos

## Abstract

Simulators are increasingly being used in medical education, but accessibility is restricted by their elevated cost. A accessible and low-cost model was developed for teaching and learning vascular sutures and anastomoses at a Basic Surgical Techniques Laboratory. Latex balloons of varying colors, polypropylene 6.0 sutures, and other materials specifically for suturing (needle holder and forceps) were used. The balloons were fixed to screws inserted into wooden boards in order to facilitate repairs. E end-to-end, end-to-side, and side-to-side anastomoses and patching were performed. Anastomosis patency was tested by injecting water into one extremity of the balloon and observing the liquid exit via the opposite extremity. The advantages observed with this training model for anastomoses were malleability, resistance to passage of the suture, and the fact that it is inorganic. Latex balloons are an inexpensive option that are non‑perishable and offer prolonged use for teaching and practice of arterial sutures and anastomoses.

## INTRODUÇÃO

 Na disciplina de Bases das Técnicas dos Procedimentos Cirúrgicos e Anestésicos de uma universidade, os alunos do quarto período da graduação em medicina iniciam o aprendizado dos materiais cirúrgicos, tais como instrumentais e fios, e das técnicas cirúrgicas, tais como nós, suturas e anastomoses. A partir de uma autoavaliação em que os alunos, monitores e professores avaliaram a si mesmos em relação ao aprendizado nas aulas da disciplina, e de um questionário de avaliação da disciplina respondido pelos alunos da graduação, tornou-se evidente a deficiência no ensino teórico-prático do tema anastomoses. Assim, propusemos um aprofundamento desse tema, iniciando por anastomoses vasculares, em virtude da importância desse tipo de anastomose em toda especialidade cirúrgica e não somente na cirurgia vascular ou cardiovascular. 

 O uso de simuladores na educação médica tem sido cada vez mais frequente. Contudo, simuladores são pouco acessíveis para a maioria das faculdades e hospitais do Brasil, devido ao seu elevado custo. Na literatura, é descrita a utilização de materiais sintéticos, como silicone e luvas de borracha, de tecidos animais, tecidos vegetais e prática em pequenos animais (porco, ratos e coelhos) para o aprendizado e desenvolvimento da habilidade em anastomose vasculares 1 ^-^ 5 . No entanto, o uso de modelos animais é o principal ponto adverso levantado pelo público e pelos próprios alunos, que questionam sacrificar animais para fins de aprendizado 6 . As estritas leis dos Comitês Institucionais de Cuidados e Utilização de Animais também dificultam o processo de utilização desses modelos animais na educação médica, contribuindo para a necessidade do desenvolvimento de alternativas a esses modelos. 

 O objetivo desse artigo foi descrever a criação de um modelo com balões de látex para ensino e treinamento de suturas e anastomoses vasculares no laboratório de disciplina de Bases das Técnicas Cirúrgicas de uma Universidade. 

## MATERIAIS E MÉTODOS

 Para construção desse modelo, foram utilizados sete balões de látex de 27 x 0,9 cm de cores variadas, quatro placas de madeira, nove parafusos, fio cirúrgico de polipropileno cardiovascular 6.0 com duas agulhas de 1,3 cm ( Figura 1 ) e outros materiais específicos para sutura vascular (porta-agulhas, pinça Kelly protegida com látex e pinça de preensão atraumática). 

**Figura 1:** Materiais para a construção do modelo: balões de látex de cores variadas (A); parafusos (B); placas de madeira (C); fio de polipropileno 6.0 com duas agulhas (D).

 As extremidades e o excedente dos balões foram utilizados para recobrir os parafusos ( Figura 2 ). Isso permitiu a fixação do modelo à placa de madeira e a economia de material, pois, por não ter sido descartado, o comprimento extra pode ser utilizado em outro momento. 

**Figura 2:** Preparação da placa de madeira com parafusos.

 A sutura do *patch* e as anastomoses dos balões foram realizadas conforme os princípios básicos das anastomoses vasculares descritos por Carrel 7 ^-^ 9 , Guthrie 10 e Rutherford 11 , com utilização de dois fios de polipropileno com duas agulhas em cada um deles. 

 A anastomose terminoterminal foi realizada com pontos separados ( Figura 3 ) pela técnica de triangulação 12 ^,^ 13 , com três pontos equidistantes, transformando a circunferência em um triângulo. Esse tipo de anastomose é recomendado para crianças e vasos de menor calibre. Como as extremidades eram do mesmo calibre, elas não foram biseladas. Nessa técnica, os lados também podem ser aproximados com pontos tipo chuleio simples. 

**Figura 3:** Anastomose terminoterminal com pontos separados.

 A sutura do *patch* e as anastomoses terminolateral e laterolateral foram realizadas com pontos iniciais nos ângulos e fechamento das paredes anterior e posterior com sutura contínua 12 ^,^ 13 . Além disso, na anastomose terminolateral, conforme a técnica recomendada por Rutherford, a abertura do balão foi uma vez e meia maior que o calibre do balão a ser implantado e biselado com angulação de 30 a 45 graus em relação ao mesmo balão. Algumas etapas na confeção da confecção da anastomose terminolateral são demonstradas na Figura 4 . 

**Figura 4:** Confecção da anastomose terminolateral: sutura inicial aproximando os balões com pontos nos ângulos (proximal e distal) (A); sutura contínua em quatro quadrantes (B); detalhe na passagem da agulha em toda a parede do balão (C); aspecto final da anastomose (D).

 Assim, na construção desse modelo, vários tipos de anastomoses foram testados: anastomoses terminoterminal, laterolateral, terminolateral e construção de *patch,* utilizando-se quatro placas de madeira, com aspecto final demonstrado na Figura 5 . 

**Figura 5:** Aspecto final dos modelos das anastomoses confeccionadas nas quatro placas de madeira: terminoterminal (A); laterolateral (B); *patch* (C) e terminolateral (D).

 A perviedade do *patch* e das anastomoses foi testada a partir da injeção de água em uma extremidade do balão e a observação da saída desse conteúdo na extremidade oposta (anastomoses terminoterminal e laterolateral e construção de *patch*) ou na extremidade de interesse (anastomose terminolateral). 

## RESULTADOS

 Foram realizadas anastomoses vasculares (terminoterminal, laterolateral, terminolateral) em três estações diferentes, e foi realizada a construção de *patch* em uma outra estação. Em todos esses procedimentos, foram utilizados balões de látex de cores diferentes. Na confecção das suturas, observou-se resistência à passagem da agulha no balão de látex, semelhante àquela observada em artérias e veias normais, não ateroscleróticas. O material é firme, não ocorrendo laceração em nenhuma das estações. Além disso, as bordas mantiveram-se bem coaptadas com os fios e nós. A diferença de cor entre os balões utilizados em cada anastomose apresentou-se como um aspecto interessante, uma vez que facilitou a visualização da passagem dos pontos, da coaptação das bordas e do resultado final. 

 A perviedade testada foi de 100% com a instilação líquida. Na anastomose terminoterminal e na confeção do *patch*, obsevou-se que o líquido também extravasou entre os pontos. 

 O custo para a construção desse modelo, com a realização das quatro anastomoses, foi de R$ 152,00 (oito fios cirúrgicos de polipropileno 6.0, sete balões de látex, parafusos e quatro placas de madeira), sendo a maior parte destinada à aquisição dos fios para a sutura. 

## DISCUSSÃO

 A confecção de anastomoses vasculares é um procedimento realizado em diversas especialidades cirúrgicas e não somente nas cirurgias vascular e cardiovascular 14 ^,^ 15 . Na maioria das vezes, os alunos e cirurgiões iniciantes aprendem e treinam essas anastomoses diretamente em seres humanos, podendo acarretar prejuízos aos pacientes, devido ao aumento do tempo cirúrgico ou a complicações pós-operatórias, e consequente incremento de custos hospitalares 14 ^,^ 16 . Em muitos casos, isso é decorrente da rotina estabelecida há anos no serviço ou da falta de laboratórios equipados com modelos acessíveis e sintéticos nas faculdades ou em ambientes extra-hospitalares. 

 Vários estudos já demonstraram que tanto o aprendizado como o treinamento devem ser feitos de maneira laboratorial antes de serem feito no ambiente hospitalar e na prática clínica 14 ^,^ 15 ^,^ 17 . Alguns autores recomendam que o treinamento prévio em simuladores deva ser uma etapa fundamental na formação do futuro cirurgião 2 ^,^ 18 . Devido ao elevado custo dos simuladores disponíveis no mercado, alguns modelos alternativos de baixo custo foram desenvolvidos. 

 Excluindo o uso de animais vivos, a literatura descreve tipos de modelos com materiais diversos. 

 Webster & Ely 18 e Lima et al. 19 testaram a confecção de anastomose terminoterminal com fios de mononylon em tubos de silicone e mostraram que essa técnica é eficaz no treinamento inicial. 

 O uso de luvas de borracha para o treinamento de anastomoses vasculares é descrito por alguns autores, mas sem referência bibliográfica ou descrição dos métodos e resultados. 

 Achar et al. 1 descreveram um modelo experimental com traqueia e esôfago extraídos de cabeças de frango e simulou sutura arterial com a confecção de anastomose terminoterminal. Já Colpan et al. 20 desenvolveram um modelo realista para treinamento de anastomoses vasculares com carótidas de pescoço de peru, que eram perfundidas durante o procedimento. Maluf et al. 5 utilizaram o pedículo esplênico de suínos pós-mortem, que foram submetidos a esplenectomia, como um modelo alternativo para treinamento de anastomose vascular com fios de mononylon. 

 Grahem et al. 2 testaram vagem e feijão verde como modelo de baixo custo na confecção de anastomose terminoterminal com fios de polipropileno e consideraram as características do feijão verde mais apropriadas para o treinamento inicial. 

 O material utilizado em nosso estudo, balões de látex, não havia sido descrito anteriormente na literatura com essa finalidade. O balão de látex se assemelha a um vaso por ser cilíndrico (27 cm de comprimento), ter calibre de 0,9 cm com espessura delgada (menor que 1 mm), e apresentar luz interna e maleável. Além disso, se assemelha à parede de artérias e veias normais por apresentar pouca resistência à passagem da agulha do fio de polipropileno. 

 As características do látex, quanto à sua maleabilidade e resistência, favorecem o ensino da técnica dos vários tipos de anastomoses (terminoterminal, laterolateral, terminolateral e colocação de *patch*), as quais foram testadas em nosso estudo. Assim, demonstramos que a principal vantagem do modelo com balões de látex é a possibilidade de utilizá-lo para o ensino de várias técnicas de suturas vasculares, diferentemente do que é descrito na maioria dos estudos da literatura, que testaram somente anastomose terminoterminal 2 ^,^ 3 ^,^ 6 ^,^ 19 ^,^ 20 . Alguns estudos utilizaram fios de mononylon para a confecção das anastomoses 6 ^,^ 19 ^,^ 20 . Em nosso modelo, as anastomoses foram feitas com fio de polipropileno de fino calibre e com duas agulhas, para deixar o modelo mais real e contribuir para a familiarização do aluno com a técnica vascular. 

 Outra característica peculiar observada foi a diferença das cores dos balões, o que facilita a visualização das partes a serem anastomosadas, sendo assim relevante no aprendizado e entendimento da construção da anastomose pelos alunos. Como o silicone, o látex é um material inorgânico, acessível e disponível, características úteis e fundamentais em centros que, assim como o nosso, não possuem biotério ou laboratório de cirurgia experimental. 

 Duas desvantagens de nosso modelo foram observadas. A primeira foi a inexistência de uma lâmina íntima no balão. Durante as anastomoses vasculares *in vivo*, dedica-se atenção para a fixação e manutenção da união da camada íntima com a parede do vaso, a fim de evitar seu descolamento ou “flap”. Alterações nessa camada são frequentemente fatores predisponentes para complicações pós-operatórias e oclusões das anastomoses vasculares. A segunda foi o extravasamento do líquido infundido durante o teste dos pontos, tornando-o insatisfatório por não permitir avaliar se a distância entre eles está adequada. Acredita-se que isso ocorreu em virtude das próprias características do balão – maleável, elástico e inorgânico – e da consequente ausência de fatores sanguíneos da coagulação que promovem a coesão de células teciduais. Essas mesmas desvantagens também foram observadas por Grahem et al. 2 em modelo com tecidos vegetais. 

 Entretanto, acreditamos que, na curva inicial de aprendizado do aluno e até mesmo do jovem cirurgião, as vantagens se sobrepõem às desvantagens. Isso é especialmente verdadeiro no que diz respeito ao ensino da técnica, ao treinamento e à familiarização com o instrumental vascular específico, à prática de anastomose de estruturas com o uso de fios de pequeno calibre e com duas agulhas, e ao desenvolvimento progressivo da destreza e da agilidade da técnica com movimentos delicados. 

## CONCLUSÃO

 Os balões de látex são uma alternativa interessante, viável e de baixo custo, comparados aos modelos descritos anteriormente na literatura para o aprendizado da técnica e o treinamento da confecção de suturas e de diversos tipos de anastomoses vasculares pelos alunos de graduação e cirurgiões iniciantes. Suas características os fazem disponíveis e de acesso universal, contribuindo para a melhoria da educação médica.
